# Using Celery Powder in a Semi-Dry Fermented Sausage ‘Heat-Treated Sucuk’: Nitrosamine Formation, Lipid Oxidation, and Volatile Compounds

**DOI:** 10.3390/foods13203306

**Published:** 2024-10-18

**Authors:** Zeynep Feyza Yılmaz Oral, Mükerrem Kaya, Güzin Kaban

**Affiliations:** Department of Food Engineering, Faculty of Agriculture, Atatürk University, 25240 Erzurum, Türkiye; mkaya@atauni.edu.tr (M.K.); gkaban@atauni.edu.tr (G.K.)

**Keywords:** fermented sausage, heat-treated sucuk, nitrosamine, celery powder, volatile compound

## Abstract

This study investigated the effect of using celery powder (CP) as source of pre-converted nitrite (treatments: A: 150 mg/kg NaNO_2_, B: 100 mg/kg NaNO_2_ + CP as 50 mg/kg NaNO_2_ equivalent, C: 50 mg/kg NaNO_2_ + CP as 100 mg/kg NaNO_2_ equivalent, D: CP as 150 mg/kg NaNO_2_ equivalent) on the physicochemical and microbiological properties in heat-treated sucuk (HTS), a kind of semi-dry fermented sausage. The influence of cooking time (CT) on the nitrosamine formation in HTS with and without CP was also determined. The results indicated that the use of CP increased the pH value and decreased the a_w_ value. *Micrococcus/Staphylococcus* and residual nitrite were not affected by the use of CP. TBARS value varied from 0.78 to 0.90 mg MDA/kg. CP did not affect the abundance of hexanal in HTS, however, it increased the abundance of camphene. The results of PCA showed that treatments A, B, and C had similar volatile compound profiles. CP did not affect both N-nitrosodimethylamine and N-nitrosodiethylamine, but their levels increased as the CT increased. Increased CT also resulted in increased N-nitrosopiperidine (NPIP) in all treatments, but the cooking for 1 min did not cause a significant increase in treatments A, B, and C. CP leads to a significant increase in NPIP content, especially after 3 and 5 min of cooking in HTS.

## 1. Introduction

Nitrite is an additive that is involved in the formation of the characteristic color and flavor of cured meat products. Another important function of this curing agent is its antimicrobial activity against both spoilage microorganisms and foodborne pathogens. In addition, nitrite increases the shelf life of the product by delaying lipid oxidation. However, nitrite also plays an important role in the formation of carcinogenic N-nitrosamines, and therefore, cured meat products such as fermented sausages pose a major concern for consumers [1,2,3,4]. The most common N-nitrosamines in fermented sausages are N-nitrosodimethylamine (NDMA), N-nitrosopiperidine (NPIP), and N-nitrosopyrrolidine (NPYR) [5,6]. Of these three compounds, NDMA is classified as a probable carcinogen in the 2A group. In contrast, NPIP and NPYR are defined as possible carcinogens (2B) [7]. Moreover, N-nitrosomorpholine, N-Nitrosodibutylamine, N-Nitrosodiethylamine, and N-nitrosomethylethylamine were detected in fermented sausages [3,6,8].

Since nitrite, a multifunctional additive is also effective in nitrosamine formation, strategies to reduce the ingoing nitrite level or completely remove it from the formulation have been on the agenda for a long time. Consumer awareness has been very effective in this regard, and strategies to reduce ongoing nitrite levels have been developed. Studies have been focused on two alternatives: (a) the use of other preservation techniques to reduce nitrite levels (lactate, acetate, organic acids, bacteriocins, and protective cultures) [9,10,11], (b) replacing nitrite with vegetable powders or using vegetable powders entirely instead of nitrite (celery, spinach, radish, chard, and lettuce) [9,10,11,12,13,14,15]. On the other hand, the reduction of nitrite or the use of alternative nitrite sources can also be considered part of clean label applications [16].

The nitrosamine content of fermented sausages may vary from product to product [3,6,17,18]. While these products are generally consumed raw, sucuk (dry fermented sausage) and heat-treated sucuk (HTS) (a kind of semi-dry fermented sausage) are generally consumed cooked. The studies on sucuk and HTS showed that cooking significantly increases the nitrosamine content in these sausages [6,19]. Therefore, strategies to prevent the formation of nitrosamines in sucuk or HTS are of great importance. Vegetables such as celery, spinach, radish, Swiss chard, and lettuce are frequently considered alternative curing agents due to their high nitrate content. It is noted that celery products (celery powder (CP) and celery juice concentrate) have found more use as natural nitrate/nitrite sources as they do not have a negative effect on the flavor of cured meat products and also have a very low effect on product color [9,20,21]. In addition, there is limited research on the effects of vegetable-based alternatives, including celery products, as a source of nitrate or nitrite on nitrosamine formation in fermented sausages [22,23]. Yılmaz Oral [22] reported the impact of bio-converted Swiss chard powder as a nitrite source on the formation of nitrosamines in HTS. In another study, the effect of CP on nitrosamine formation in sucuk, a dry fermented sausage type, was also investigated [23]. The effect of CP on physico-chemical properties and nitrosamine formation in semi-dry fermented sausages, including HTS, has not yet been investigated. The production of HTS includes fermentation, heat treatment, and drying stages. The aim of the study was to investigate the effect of CP as an alternative curing agent in the production of HTS on the physical, chemical, and microbiological properties of the product and the formation of nitrosamine. The study also aimed to determine the effect of cooking time in the presence of CP on nitrosamine formation in HTS.

## 2. Materials and Methods

### 2.1. Material

Meat was obtained from round parts of the beef carcasses, which were rested at 4 °C for one day from Erzurum Meat and Dairy Institution (Erzurum, Türkiye). Salt, garlic, and spices were obtained from local markets, sodium nitrite was obtained from a commercial company (Merck, Darmstadt, Germany), and celery powder (CP) (Veg Stable 506, nitrite content: 2.10%) from another commercial company (Florida Food Products, Lake Mary, FL, USA). As starter cultures, *Latilactobacillus sakei* S15 (10^7^ cfu/g) [24] and *Staphylococcus xylosus* GM92 (10^6^ cfu/g) [25] were used.

### 2.2. Production of Heat-Treated Sucuk (HTS)

In the production of HTS, beef meat and beef fat (80% lean beef + 20% beef meat fat) were used. For per kg meat and fat, 20 g NaCl, 4 g sucrose, 10 g garlic, 7 g red pepper, 9 g cumin, 5 g black pepper, and 2.5 g allspice were used [26]. Based on this formulation, four different sausage batters were prepared (Table 1).

For each treatment, four kg meat + fat mixture (3.2 kg beef + 0.8 kg beef fat) were used. Sausage batters were prepared using a laboratory-type cutter (MTK 662, Mado, Dornhan, Schwarzwald, Germany), and *L. sakei* S15 and *S. xylosus* GM92 were added to the batters as starter cultures. Then, they were stuffed into collagen casings (ø 38 mm, Naturin Viscofan GmbH, Weinheim, Germany) using a laboratory-type filling machine (MTK 591, Mado, Dornhan, Schwarzwald, Germany). The sausages were subjected to fermentation at 22 ± 1 °C and 92 ± 2% relative humidity (RH) for 22–24 h in an automatic chamber (Reich, Klima-Rauchertechnik, Stuttgart, Germany) and to heat treatment, starting from 55 °C in the cooking-chamber (Mauting, Valtice, Czech Republic) with a core temperature of 64 °C. The samples were then subjected to a drying program (2 days at 18 ± 1 °C and 84 ± 2% RH). For the productions, three different carcasses as raw materials were used at three different times.

### 2.3. Cooking Procedure of HTS

After production, HTS samples belonging to each group were sliced to 0.5 cm thickness using a slicing machine (Işıklar, İstanbul, Türkiye) in order to evaluate the effect of cooking level on nitrosamine and then fried on a hot plate (Elektro-mag, İstanbul, Türkiye) at 180 °C. The four different cooking times (0 min—control, 1 min, 3 min, and 5 min) were applied.

### 2.4. Microbiological Analysis

Lactic acid bacteria and Enterobacteriaceae were determined on de Man Rogosa Sharpe Agar (MRS, Merck, Darmstadt, Germany) and Violet Red Bile Dextrose Agar (VRBD, Merck), using spread plate technique, respectively, and the counts were evaluated after anaerobic incubation (Anaerocult A, Merck) (at 30 °C for two days). *Micrococcus/Staphylococcus* counts were determined by the spread plate technique on Mannitol Salt Phenol Red Agar (MSA, Merck). MSA plates were aerobically incubated at 30 °C for two days. After incubation, catalase-positive colonies were taken into account, and their number was determined [27].

### 2.5. Physicochemical Analysis

#### 2.5.1. Determination of pH and a_w_

After a 10 g sample was added to 100 mL of distilled water, it was homogenized using an ultra-turrax (IKA T25, Werke GmbH, Staufen, Germany), and the measurement was performed using a pH meter. The a_w_ value was performed using an a_w_ meter (TH-500, Novasina, Lachen, Switzerland) [18].

#### 2.5.2. Determination of TBARS

A 2 g sample was mixed with 12 mL trichloroacetic acid solution (7.5%) and homogenized. After filtration (Whatman 1, Merck, Germany), 3 mL of filtrate was added to 3 mL of thiobarbituric acid solution (0.02 M). This mixture was kept in a boiling water bath for 40 min and then in cold water for 5 min. After centrifugation (5 min at 2000× *g*) (Thermo MR23I, Waltham, MA, USA), the absorbance of samples was determined at 530 nm in a spectrophotometer (Thermo, Waltham, MA, USA). TBARS value was provided as mg MDA/kg sample [28].

#### 2.5.3. Determination of Residual Nitrite

The analysis was performed according to the method provided by NMKL [29]. The amount of residual nitrite was determined using HPLC/DAD (Agilent 1100, Santa Clara, CA, USA). The different concentrations (10–40 mg/L) were spiked into samples to calculate mean recovery and relative standard deviations, and LOD and LOQ values were calculated.

#### 2.5.4. Determination of Color Values

HTS slices (0.5 cm) were used to measure the color parameters. The values of L (lightness), a* (red-green component), and b* (yellow-blue component) of HTS were performed using the Chroma Meter (Konica CR-400, Minolta, Osaka, Japan) [26].

#### 2.5.5. Determination of N-Nitrosamines

A 10 g sample was added to 0.1 M NaOH, and the mixture was sonicated for 15 min in an ultrasonic bath (RK 512 H, Bandelin Electronic, Berlin, Germany). After that, methanol (20 mL) was added and homogenized using ultra-turrax (T25D, IKA, Werke, Staufen, Germany). After centrifuging (10,000 rpm at 4 °C), the samples were filtered using glass microfiber. A total of 15 mL of filtrate and 5 mL of 20% NaCl were transferred to the ChemElut column (Agilent, USA). After adding dichloromethane to the mixture, it was concentrated using a Kuderna Danish apparatus and evaporated under a nitrogen stream at 40 °C [30]. The nitrosamine content was determined using GC/MS (6890 N/5973 N, Agilent Technologies, Santa Clara, CA, USA) in selective ion mode.

The limit of quantification (LOQ) and limits of detection (LOD) of the N-nitrosamines were verified by different concentrations (from 0.5 to 20 μg/L) of the standard mix.

#### 2.5.6. Volatile Compound Analysis

Homogenized samples of 5 g were weighed into 40 mL vials, and the vials were kept in a thermal block (Supelco, Bellefonte, PA, USA) for 1 h at 30 °C. Then, the fiber (carboxen/polydimethylsiloxane, Supelco, USA) was injected into the vial, and the extraction was carried out for 1 h at 30 °C. After extraction, the fiber was desorbed (for 6 min at 250 °C) to gas chromatography/mass spectrometry (Agilent Technologies, Santa Clara, CA, USA). The oven temperature was initially set at 40 °C for 5 min and then gradually increased to 210 °C and kept for 12 min. The carrier gas in the system was helium, and the column was DB-624 (60 m × 0.250 mm × 1.40 µm, Agilent Technologies, Santa Clara, CA, USA). The identification was carried out using mass spectrometry libraries (Nist, Flavor, and Wiley), and the Kovats index was determined using the standard mix (Supelco 44585-U, Bellefonte, PA, USA), and standard substances were used for the identification of compounds. The results were presented as Au × 10^6^ [31].

### 2.6. Statistical Analysis

The study was conducted in four different treatments (treatments: A: 150 mg/kg NaNO_2_, B: 100 mg/kg NaNO_2_ + CP as 50 mg/kg NaNO_2_ equivalent, C: 50 mg/kg NaNO_2_ + CP as 100 mg/kg NaNO_2_ equivalent, D: CP as 150 mg/kg NaNO_2_ equivalent). The experiments were conducted in a randomized complete block design with three replications. In each experiment, different raw materials were used, and production was carried out at different times. As nitrosamine analyses were performed on raw and cooked samples, cooking time was considered a factor in addition to the treatment factor, and the experiments were performed in a 4 × 4 (treatment × cooking time) factorial design. Data were analyzed by analysis of variance using a general linear model with treatment (use of CP) as the main effect and replicates as a random effect. Cooking time was also evaluated as a main effect for nitrosamines analysis. Duncan’s multiple range test was performed to define differences between means (SPSS 24, Chicago, IL, USA). Principal component analysis (PCA) was carried out using Minitab 19 to define the relationship between treatments and volatile compounds. Cluster analysis of the heat map was also performed using chioplot to determine the relationship between nitrosamines and treatments cooked at different times (https://www.chiplot.online, accessed on 10 August 2024).

## 3. Results and Discussion

### 3.1. pH, a_w,_ and TBARS

Celery powder (CP) usage had a very significant effect (*p* < 0.01) on pH. The treatment D (containing only CP as a curing agent) showed the highest pH value (Table 2). It is assumed that this result is due to the alkaline character of the CP used (pH 8.0–10.0). It has also been reported that CP increases the pH in cured restructured cooked hams, and this increase is due to the pH of the CP used [32]. Similarly, in another study conducted on ham, it was reported that the pH value increased with celery juice concentrate [33]. On the other hand, in some studies conducted on HTS, it was determined that as the concentration of Swiss chard powder increased, the pH value also increased [22,34]. Differences were also determined between the treatments in terms of a_w_ value. The lowest a_w_ value was determined in treatment D. This result is probably due to the water retention properties of CP. In previous studies, it has also been shown that the addition of vegetable powders (celery and Swiss chard) decreases the a_w_ value in sucuk and HTS [22,23]. CP usage had a very significant effect (*p* < 0.01) on TBARS values of HTS. As seen in Table 2, the use of CP caused an increase in TBARS value, an indicator of lipid oxidation. Similarly, it was reported that Swiss chard powder causes an increase in TBARS value in HTS [22]. Sindelar et al. [35] also indicated that the use of CP in ham promotes lipid oxidation. Factors such as formulation, raw material properties, degree of mincing of meat and fat, salt, nitrite, spices, antioxidants, and pH significantly affect lipid oxidation in fermented sausages [36]. In our study, however, in all treatments, the TBARS value was lower than the maximum limit value (1 mg MDA/kg) (Table 2) [37].

### 3.2. Instrumental Color

The L* and b* values of HTS were not affected by the use of synthetic nitrite and/or CP as a nitrite source in production (*p* ˃ 0.05). In contrast, the use of CP had an effect on a* value at *p* < 0.05 level (Table 2). The lowest a* value was obtained in HTS cured only with CP (treatment D). Similarly, CP was reported to give a lower a* value than synthetic nitrite in cold smoked sausage, and it was emphasized that this result was due to green celery pigments, which give a specific color to vegetable powders [38]. As seen in Table 2, there was no significant difference in terms of a* value between the treatments containing 150, 100, or 50 mg/kg synthetic nitrite (treatments A, B, and C). This result shows that the 50 mg/kg of synthetic nitrite is sufficient in terms of a* value. In fact, it is stated that even the presence of less than 25 mg/kg of nitrite in cured meat products is sufficient for color formation [39].

### 3.3. Residual Nitrite

The mean recovery of nitrite ranged from 98.84% to 104.87%, with relative standard deviations ranging from 0.63% to 1.46%. The coefficient of the regression line, LOD, and LOQ values for nitrite were determined as 0.9995, 1.01 mg/kg, and 3.06 mg/kg, respectively. The use of CP in production on HTS did not show a significant effect on residual nitrite (*p* > 0.05), and residual nitrite varied between 12.93 and 13.80 mg/kg (Table 2). Similarly, no significant effect on residual nitrite in sucuk was found for CP (bio-converted) and beet powder [12,23]. Another study also reported that Swiss chard powder did not affect the residual nitrite of HTS [22]. Similarly, celery had no significant effect on the residual nitrite of emulsion-type sausages, as reported by Jung et al. [40]. On the other hand, Wang et al. [41] reported that *L. sakei* significantly reduced the amount of residual nitrite in Chinese sausages. In the present study, *L. sakei* S15, used as a starter culture, caused acidification during fermentation (Table 2). The acidification is thought to accelerate the conversion of nitrite to nitric oxide, thus reducing the residual nitrite [3].

### 3.4. Lactic Acid Bacteria, Micrococcus/Staphylococcus, and Enterobacteriaceae

The effect of using CP as a source of nitrite on the microbiological properties of HTS is provided in Table 3. The use of CP reduced the number of lactic acid bacteria (LAB). However, the difference between treatment A and treatment D was found to be statistically significant (*p* < 0.05). In HTS production, there are three main stages (fermentation, heat treatment, and drying). Heat treatment (core temperature 60–68 °C) applied after fermentation causes microbial reduction [22,26]. In the current study, the heat treatment was based on an internal temperature of 64 °C. The count of LAB in the final product varied between 2 and 3 log cfu/g. However, a lower number of LAB has been reported at an internal temperature of 68 °C [26]. *Staphylococcus xylosus* GM92 was added to HTS batters with 10^6^ cfu/g. After production, there was no significant difference in the number of *Micrococcus/Staphylococcus* among the treatments (*p* ˃ 0.05) (Table 3). CP was also reported to have no effect on *Micrococcus/Staphylococcus* in sucuk [23]. *Micrococcus/Staphylococcus* number in the final product was determined at the level of 3 log cfu/g for all treatments. These findings are similar to those obtained in other studies on HTS [22,26]. Due to the application of heat treatment, the number of Enterobacteriaceae in the final product was determined to be < 2 log cfu/g in all treatments (Table 3).

### 3.5. Volatile Compounds

HTS has a very complex structure. It contains high levels of protein and fat, as well as some other minor components and spices that are added during production. Furthermore, heat treatment at 60–68 °C can be effective on the volatile compounds of HTS [26]. In the HTS groups, a total of 49 volatile compounds were identified, belonging to 8 different chemical groups, including 5 alcohols, 5 sulfur compounds, 5 aliphatic hydrocarbons, 2 ketones, 5 aromatic hydrocarbons, 5 aldehydes, 2 esters, and 20 terpenes (Table 4). As can be seen from the results, terpenes are a class of compounds that play an important part in the volatile compound profile of HTS. In another study conducted on HTS, it was also reported that terpenes are a significant part of the volatile profile of HTS [26]. Of the 49 compounds identified, only 4 compounds (ethanol, allyl mercaptan, α-terpinolene, and camphene) were affected by the addition of CP. The lowest abundance of ethanol was obtained in treatment A. However, the difference between the means of treatment A and B was not found to be statistically significant (Table 4). Regarding allyl mercaptan, treatment A also showed higher abundance compared to the other treatments (B, C, and D) (*p* < 0.05). Aldehydes, which are the typical aroma source in fermented sausages and ripened meats due to their low threshold values, can be formed as a result of lipid oxidation and amino acid catabolism [26]. Five aldehydes were found in all treatment groups. CP had no significant effect on these compounds, including hexanal (*p* ˃ 0.05). Of the identified terpenes, only α-terpinolene and camphene were affected by the addition of CP. The highest value for α-terpinolene was found in treatment A, which was only cured with synthetic nitrite (*p* < 0.05). This treatment also provided the lowest mean value for camphene (*p* < 0.05). The abundance of camphene increased as CP increased, but no statistically significant difference was observed among treatments containing CP (*p* ˃ 0.05) (Table 4). The flavor profile of celery can vary depending on factors such as genotype, season, part of the plant consumed, geographical region, and stage of harvest. On the other hand, it is reported that the processes applied in the production of CP have an effect on the volatile compound profile and that it has an aromatic, slightly camphoraceous odor and taste [42]. In this study, it was determined that the camphene content increased depending on the amount of CP in HTS (Table 4).

**Figure 1:**
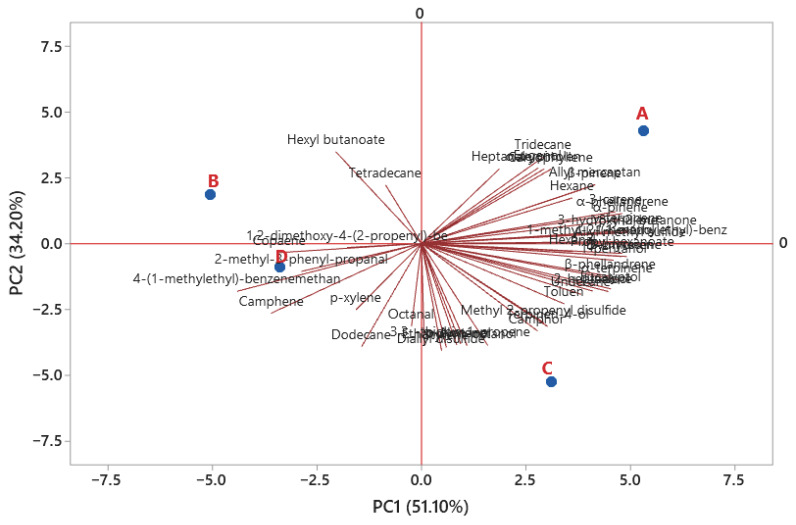
Principal component analysis of the relationships between treatments and volatile compounds. A: 150 mg/kg NaNO_2_, B: 100 mg/kg NaNO_2_ + CP as 50 mg/kg NaNO_2_ equivalent, C: 50 mg/kg NaNO_2_ + CP as 100 mg/kg NaNO_2_ equivalent, D: CP as 150 mg/kg NaNO_2_ equivalent.

In PC1, explaining 51.10% of the variance, treatment A and treatment C were placed on the positive side, while treatment B and treatment D were placed on the negative side in PC1 (Figure 1). Volatile compounds were also concentrated on the positive side of PC1, and more volatile compounds showed close association with treatments A and C. In PC2, explaining 34.20% of the variance, treatments A and B were placed on the positive side, and treatments C and D were placed on the negative side. There is no relationship between treatments A and D in either PC1 or PC2 (Figure 1). In other words, these groups did not show any similarity to each other in terms of volatile compounds. This result shows that the use of CP with 50 or 100 mg/kg synthetic nitrite is necessary to obtain a volatile compound profile similar to that of synthetic nitrite.

### 3.6. Nitrosamines

The coefficient of the regression line for NPIP, NPYR, NDBA, NMEA, NDMA, NDEA, and NDPA was 0.9999. LOD for NPIP, NPYR, NDBA, NMEA, NDMA, NDEA, and NDPA were determined as 0.32, 0.36, 0.38, 0.42, 0.32, 0.44, and 0.15 μg/kg, respectively. LOQ for NPIP, NPYR, NDBA, NMEA, NDMA, NDEA, and NDPA were determined as 0.98, 1.09, 1.15, 1.27, 0.98, 1.34, and 0.46 μg/kg. The effects of using CP as a source of nitrite and cooking time on the levels of nitrosamine of HTS are provided in Table 5. N-nitrosodimethylamine (NDMA) is one of the most frequently detected nitrosamines in HTS and sucuk [6,18]. This nitrosamine belongs to the group of probable human carcinogens (Group 2A) [7]. The CP had no significant effect on NDMA (*p* ˃ 0.05). Similarly, it has been reported that this natural curing agent has no effect on NDMA formation in sucuk [23]. In addition, another study reported that Swiss chard powder had no effect on NDMA levels in HTS [22]. NDMA content was not affected by the use of CP in HTS production. This nitrosamine also belongs to group 2A carcinogens [7]. No significant effect on NDEA was also reported when Swiss chard powder was used as a nitrite source in HTS [22]. As shown in Table 5, the NDMA and NDEA content of HTS increased with increasing cooking time. However, these nitrosamines were not affected by the CP × CT interaction (*p* ˃ 0.05). Other studies on sucuk and HTS have also found that the NDMA and NDEA content increased with the degree or time of cooking [6,19,22,23]. Cooking method and degree play an important role in the formation of nitrosamines in meat products. Heat treatment may accelerate reactions in the meat, including nitrosation. It leads to the release of nitrogen oxides or other lipid-bound nitrosating agents [6,43].

**Figure 2:**
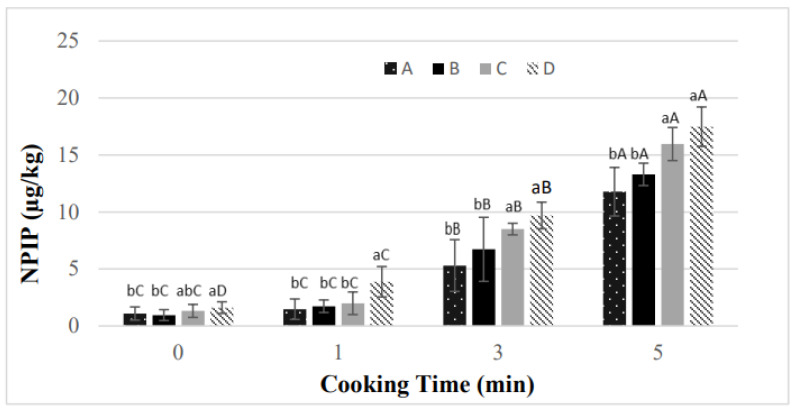
The effects of the interaction of using CP and cooking time on the level of NPIP. a–c: Different small letters indicate significant differences between treatments for cooking time. A,B: Different capitals indicate significant differences between cooking times for treatment. A: 150 mg/kg NaNO_2_, B: 100 mg/kg NaNO_2_ + CP as 50 mg/kg NaNO_2_ equivalent, C: 50 mg/kg NaNO_2_ + CP as 100 mg/kg NaNO_2_ equivalent, D: CP as 150 mg/kg NaNO_2_ equivalent.

The use of CP in HTS production had a very significant impact on N-nitrosopiperidine (NPIP). As the amount of CP added to the HTS batter increased, the NPIP content increased, and the highest main NPIP content was found in the HTS cured with only CP (Table 5). It has been reported that the use of CP in sucuk production also increases the NPIP content [23]. However, another study showed that Swiss chard powder had no significant effect on the NPIP level of HTS [22]. It is thought that these differences in results may be related to the chemical composition of the vegetable powders. Black pepper is a good source of NPIP [44,45]. It is stated that the amount of NPIP increases as its amount in fermented sausage formulation increases [46]. Black pepper is a significant spice in the production of HTS and sucuk [19,23]. Piperine (1-piperol-piperidine) and piperidine found in this spice are the main precursors of NPIP [47,48]. Piperidine is also present in celery [49]. The present study showed that the high levels of NPIP in the treatments added to CP may be related to the piperidine content of CP. As shown in Table 5, the NPIP content increased with increasing cooking time. Treatment A provided the lowest NPIP value, while the 5 min cooking application provided the highest NPIP value. Other studies conducted on sucuk and HTS have also reported that NPIP content increases as cooking time increases [6,19,22,23]. However, as shown in Figure 2, a further increase in NPIP levels was observed with increasing cooking time in the presence of CP. Treatment D (cured with CP only) showed higher levels than the other treatments after 1 min of cooking. At both 3 and 5 min cooking, treatments C and D had higher NPIP levels than the other treatments. No significant increase in NPIP was detected after 1 min of cooking in the group cured with synthetic nitrite (treatment A). Similarly, results were observed in treatments B and C. However, NPIP content increased linearly as time progressed in treatment D. An increase was observed after 3 min in treatment C. According to these results, treatments A and B showed a similar trend during cooking (Figure 2).

**Figure 3:**
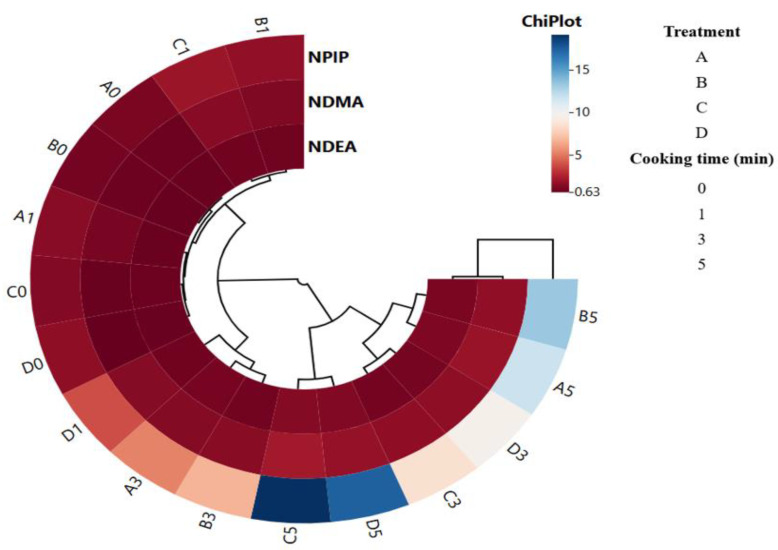
Cluster analysis of heat map showing the relationship between nitrosamine and treatments cooked at different times.

For nitrosamines, two clusters were formed, and NPIP was located in a different cluster than NDMA and NDEA (Figure 3). Although the prolonged cooking time in all groups caused differences in nitrosamine content, these differences were not remarkable in NDEA and NDMA. On the other hand, NPIP content was more affected by cooking time. There were two main clusters depending on the cooking time. The first cluster was divided into two clusters within itself. The first cluster included uncooked (0 min) samples of all treatments and 1 min cooked samples of treatments A, B, and C. The second cluster included treatments A and B cooked for 3 min and samples of treatment D cooked for 1 min. The second main cluster is divided into three clusters. In treatments C and D, 5 min cooking application was in the same cluster and showed higher NPIP values. In addition, samples cooked for 5 min in treatments A and B were in the same cluster, and 3 min samples from treatments D and C were in the same cluster and showed similarity to each other (Figure 3). These results show that when cooking is applied to treatment D, which was produced using only celery powder as a curing agent, the NPIP content increases to a greater extent than the other groups. This situation is thought to be due to the precursors contained in CP for NPIP formation.

## 4. Conclusions

The use of CP as a curing agent alone in the production of HTS can cause significant differences in the characteristic properties of the product. These differences in the profile of volatile compounds were observed to be quite significant. On the other hand, the use of CP alone or together with synthetic nitrite did not cause a significant change in the residual nitrite level. In terms of NDMA and NDEA, the use of CP does not cause a difference compared to synthetic nitrite. However, the NPIP content increases as the CP ratio increases. Moreover, this increase is greater when the cooking time is increased. As a result, the use of CP alone or together with synthetic nitrite in HTS production does not reduce the risk of nitrosamines.

## Figures and Tables

**Table 1:** Synthetic nitrite and/or nitrite derived from celery powder (CP) as curing agent for treatment.

Treatment	Curing Agent *
A	150 mg/kg NaNO_2_
B	100 mg/kg NaNO_2_ + CP as 50 mg/kg NaNO_2_ equivalent
C	50 mg/kg NaNO_2_ + CP as 100 mg/kg NaNO_2_ equivalent
D	CP as 150 mg/kg NaNO_2_ equivalent

* Calculated based on meat and fat.

**Table 2:** The effect of using celery powder (CP) as a source of nitrite on the physicochemical properties of HTS (mean± standard deviation).

Treatment	N	pH	a_w_	TBARS (mg MDA/kg)	L*	a*	b*	Residual Nitrite (mg/kg)
A	6	4.99 ± 0.10 d	0.933 ± 0.004 a	0.78 ± 0.05 c	41.04 ± 0.14	12.51 ± 0.88 a	9.79 ± 1.09	13.80 ± 2.02
B	6	5.07 ± 0.12 c	0.927 ± 0.003 b	0.82 ± 0.05 bc	39.88 ± 1.06	12.53 ± 1.12 a	9.28 ± 0.77	12.93 ± 0.86
C	6	5.14 ± 0.18 b	0.926 ± 0.001 b	0.88 ± 0.06 ab	39.86 ± 1.74	12.66 ± 0.86 a	9.58 ± 0.78	13.38 ± 1.11
D	6	5.30 ± 0.10 a	0.921 ± 0.002 c	0.90 ± 0.05 a	40.63 ± 1.52	11.66 ± 0.89 b	9.34 ± 0.95	13.60 ± 1.02
Significance		**	**	**	NS	*	NS	NS

a–d: Any two means in the same column having the same letters are not significantly different (*p* > 0.05), **: *p* < 0.01, *: *p* < 005, NS: not significant, A: 150 mg/kg NaNO_2_, B: 100 mg/kg NaNO_2_ + CP as 50 mg/kg NaNO_2_ equivalent, C: 50 mg/kg NaNO_2_ + CP as 100 mg/kg NaNO_2_ equivalent, D: CP as 150 mg/kg NaNO_2_ equivalent.

**Table 3:** The effect of using celery powder as a source of nitrite on the lactic acid bacteria, *Micrococcus/Staphylococcus* and Enterobacteriaceae of HTS (log cfu/g) (mean± standard deviation).

Treatment	N	Lactic Acid Bacteria	*Micrococcus/* *Staphylococcus*	Enterobacteriaceae
A	6	3.18 ± 0.30 a	3.29 ± 0.20	<2
B	6	2.94 ± 0.29 ab	3.17 ± 0.15	<2
C	6	2.90 ± 0.11 ab	3.27 ± 0.25	<2
D	6	2.72 ± 0.22 b	3.14 ± 0.09	<2
Significance		*	NS	NS

a–b: Any two means in the same column having the same letters are not significantly different (*p* > 0.05). *: *p* < 005, NS: not significant. A: 150 mg/kg NaNO_2_, B: 100 mg/kg NaNO_2_ + CP as 50 mg/kg NaNO_2_ equivalent, C: 50 mg/kg NaNO_2_ + CP as 100 mg/kg NaNO_2_ equivalent, D: CP as 150 mg/kg NaNO_2_ equivalent.

**Table 4:** The effect of using celery powder as a source of nitrite on volatile compounds of HTS (Au × 10^6^) (mean ± standard deviation).

Volatile Compounds	Treatment (T)					Sig.
	KI R	A	B	C	D	
Alcohols						
Ethanol	539 a	17.34 ± 5.85 c	19.64 ± 14.68 cb	45.12 ± 14.11 a	38.61 ± 7.41 ab	*
1-pentanol	821 b	0.84 ± 0.17	0.68 ± 0.12	0.86 ± 0.32	0.63 ± 0.10	NS
1-hexanol	930 a	1.93 ± 0.08	1.50 ± 0.32	1.85 ± 0.41	1.45 ± 0.23	NS
1-octanol	1127 b	1.13 ± 1.16	1.05 ± 0.91	3.28 ± 3.37	1.77 ± 0.41	NS
4-(1-methylethyl)-benzenemethanol	1380 c	8.16 ± 2.74	10.45 ± 2.52	9.69 ± 2.26	10.08 ± 3.00	NS
Sulfur Compounds						
Allyl mercaptan	574 a	334.52 ± 28.42 a	208.40 ± 50.56 b	231.43 ± 35.21 b	189.42 ± 56.43 b	*
Allyl methyl sulfide	730 b	24.84 ± 21.14	17.02 ± 9.15	23.16 ± 17.21	18.58 ± 8.39	NS
3,3′-thiobis-1-propene	888 b	37.44 ± 4.98	36.58 ± 3.70	45.66 ± 1.21	42.67 ± 3.45	NS
Methyl 2-propenyl disulfide	958 c	12.30 ± 4.38	10.51 ± 2.59	14.19 ± 1.65	12.77 ± 1.45	NS
Diallyl disulfide	1138 a	49.95 ± 10.82	52.70 ± 8.96	68.63 ± 28.42	59.47 ± 10.28	NS
Aliphatic Hydrocarbons						
Hexane	600 a	4.72 ± 3.16	1.58 ± 0.74	2.56 ± 1.63	3.20 ± 3.48	NS
Undecane	1100 a	1.23 ± 0.40	1.16 ± 0.38	1.32 ± 0.44	1.12 ± 0.45	NS
Dodecane	1200 a	4.35 ± 0.77	5.25 ± 1.54	6.19 ± 2.84	5.69 ± 1.05	NS
Tridecane	1300 a	6.41 ± 6.49	3.37 ± 1.78	2.73 ± 0.46	3.25 ± 0.43	NS
Tetradecane	1400 a	3.27 ± 1.12	2.58 ± 0.77	2.08 ± 0.73	3.54 ± 0.97	NS
Ketones						
3-hydroxy-2-butanone	779 a	6.63 ± 2.16	4.24 ± 1.21	5.83 ± 2.41	4.86 ± 2.60	NS
2-heptanone	946 a	1.02 ± 0.15	0.77 ± 0.06	1.08 ± 0.10	0.96 ± 0.31	NS
Aromatic Hydrocarbons						
Toluen	796 a	1.54 ± 0.22	1.29 ± 0.13	1.65 ± 0.41	1.55 ± 0.50	NS
p-xylene	898 c	0.80 ± 0.23	1.15 ± 1.05	1.18 ± 0.62	0.89 ± 0.32	NS
Styrene	935 c	1.11 ± 0.10	1.24 ± 0.66	1.62 ± 0.96	1.27 ± 0.15	NS
1-methyl-2-(1-methylethyl)-benzene	1072 c	211.46 ± 34.86	187.21 ± 5.30	206.55 ± 51.13	186.33 ± 28.21	NS
1,2-dimethoxy-4-(2-propenyl)-benzene	1434 c	5.51 ± 2.85	5.31 ± 0.80	5.31 ± 3.0	6.61 ± 1.30	NS
Aldehydes						
Hexanal	850 a	2.75 ± 0.97	2.07 ± 0.47	2.58 ± 0.85	2.64 ± 0.38	NS
Heptanal	955 a	4.08 ± 1.42	3.99 ± 1.64	3.81 ± 0.76	3.65 ± 0.13	NS
Octanal	1051 a	2.17 ± 0.67	2.15 ± 0.42	2.32 ± 0.33	2.36 ± 0.65	NS
Nonanal	1142 c	7.55 ± 3.21	7.48 ± 0.88	8.38 ± 2.66	8.01 ± 1.52	NS
2-methyl-3-phenyl-propanal	1333 c	94.07 ± 57.67	101.77 ± 29.88	100.81 ± 48.73	133.13 ± 37.61	NS
Esters						
Propyl hexanoate	1151 c	4.30 ± 1.79	2.99 ± 1.03	4.26 ± 1.43	2.80 ± 1.13	NS
Hexyl butanoate	1215 c	7.34 ± 1.63	7.31 ± 0.44	5.24 ± 3.58	7.36 ± 2.91	NS
Terpenes						
α-pinene	950 a	5.78 ± 3.16	3.39 ± 0.49	4.70 ± 2.61	4.07 ± 1.05	NS
β-thujene	994 b	4.60 ± 1.63	2.76 ± 0.86	3.99 ± 1.73	3.11 ± 1.10	NS
β-pinene	996 b	41.09 ± 33.16	17.91 ± 4.54	22.98 ± 8.32	20.16 ± 7.28	NS
β-myrecene	998 b	39.64 ± 2.29	29.33 ± 5.73	38.49 ± 15.11	32.89 ± 4.64	NS
α-phellandrene	1047 b	15.57 ± 4.12	9.89 ± 1.11	12.74 ± 3.84	10.35 ± 0.33	NS
3-carene	1052 b	12.41 ± 4.80	6.81 ± 1.28	9.48 ± 1.62	8.35 ± 0.39	NS
α-terpinene	1062 c	3.52 ± 0.34	2.66 ± 0.61	3.71 ± 1.23	2.91 ± 0.24	NS
D-Limonene	1080 b	26.18 ± 3.57	23.34 ± 3.79	26.22 ± 4.79	22.92 ± 0.33	NS
β-phellandrene	1083 b	6.02 ± 1.75	5.17 ± 0.86	6.33 ± 2.31	5.05 ± 0.44	NS
Eucalyptol	1088 b	5.02 ± 0.56	4.15 ± 0.47	5.31 ± 0.58	4.50 ± 0.56	NS
γ-terpinene	1102 c	141.57 ± 19.46	90.46 ± 18.63	124.11 ± 48.19	98.14 ± 18.64	NS
α-terpinolen	1105 c	2.26 ± 0.04 a	1.12 ± 0.41 b	1.11 ± 0.33 b	1.50 ± 0.27 b	*
p-cymene	1147 c	2.67 ± 2.31	2.52 ± 2.20	5.04 ± 1.35	4.16 ± 0.46	NS
Linalool	1161 a	41.72 ± 16.94	35.22 ± 2.93	44.67 ± 14.97	37.09 ± 4.60	NS
Camphor	1230 c	1.56 ± 0.59	1.30 ± 0.36	2.01 ± 0.73	1.64 ± 0.50	NS
Terpinen-4-ol	1233 b	3.92 ± 1.20	3.67 ± 0.51	4.65 ± 2.50	3.78 ± 0.67	NS
Camphene	1362 b	1.51 ± 0.71 b	2.91 ± 0.78 a	2.99 ± 0.77 a	3.60 ± 1.00 a	*
Copaene	1432 b	1.89 ± 1.12	2.32 ± 0.93	1.93 ± 0.90	3.39 ± 2.09	NS
Eugenol	1456 b	1.67 ± 0.80	1.36 ± 0.68	1.35 ± 0.39	1.47 ± 0.53	NS
Caryophyllene	1490 c	7.40 ± 3.33	5.75 ± 0.90	5.76 ± 0.79	6.22 ± 0.89	NS

KI: Kovats index calculated for DB-624 capillary column (60 m × 0.25 mm × 1.4 μm) installed on a GC/MS. R: reliability of identification; a: mass spectrum and retention time identical with an authentic sample; b: Kovats index from the literature in accordance and mass spectrum; c: tentative identification using mass spectrum, *: *p* < 0.05, NS: not significant.

**Table 5:** The effects of using celery powder as a source of nitrite and cooking time on the nitrosamine levels of HTS (µg/kg) (mean ± standard deviation).

Treatment (T)	N	NDMA	NDEA	NPIP
A	24	1.27 ± 0.52	0.90 ± 0.27	4.91 ± 4.65 d
B	24	1.29 ± 0.42	0.88 ± 0.22	5.68 ± 5.23 c
C	24	1.39 ± 0.51	0.93 ± 0.20	6.95 ± 6.11 b
D	24	1.37 ± 0.51	0.96 ± 0.28	8.16 ± 6.38 a
Significance		NS	NS	**
**Cooking Time (min) (CT)**	**N**			
0	24	0.72 ± 0.22 d	0.70 ± 0.12 d	1.24 ± 0.56 d
1	24	1.29 ± 0.27 c	0.82 ± 0.15 c	2.27 ± 1.33 c
3	24	1.53 ± 0.30 b	0.97 ± 0.14 b	7.55 ± 2.48 b
5	24	1.80 ± 0.30 a	1.18 ± 0.24 a	14.63 ± 2.73 a
Significance		**	**	**
T × CT		NS	NS	**

a–d: Any two means in the same column having the same letters in the same section are not significantly different (*p* > 0.05). **: *p* < 0.01, NS: not significant. A: 150 mg/kg NaNO_2_, B: 100 mg/kg NaNO_2_ + CP as 50 mg/kg NaNO_2_ equivalent, C: 50 mg/kg NaNO_2_ + CP as 100 mg/kg NaNO_2_ equivalent, D: CP as 150 mg/kg NaNO_2_ equivalent.

## Data Availability

The original contributions presented in the study are included in the article, further inquiries can be directed to the corresponding author.
